# Memory Effect on a LDH/zeolite A Composite: An XRD In Situ Study

**DOI:** 10.3390/ma14092102

**Published:** 2021-04-21

**Authors:** Breno G. P. Bezerra, Lindiane Bieseki, Mariele I. S. de Mello, Djalma R. da Silva, Cristiane B. Rodella, Sibele Pergher

**Affiliations:** 1 Molecular Sieves Laboratory LABPEMOL, Chemistry Institute, Federal University of Rio Grande do Norte UFRN, Natal 59078-970, RN, Brazil; brenogpb@gmail.com (B.G.P.B.); lindiane.bieseki@gmail.com (L.B.); mellomariele@gmail.com (M.I.S.d.M.); djalmarib@gmail.com (D.R.d.S.); 2 Brazilian Synchrotron Laboratory (LNLS), Brazilian Research Center of Materials and Energy (CNPEM), Campinas 13083-100, SP, Brazil; cristiane.rodella@gmail.com

**Keywords:** LDH composite, zeolite A, memory effect, XRD, in situ experiments

## Abstract

In this memory effect study, hydrotalcite-type compounds in the lamellar double hydroxide-like (LDH)/zeolite A composite material were analyzed using X-Ray Diffration XRD) in situ experiments. Three samples were analyzed: Al,Mg-LDH, Al,Mg-LDH/ZA composite, and a physical mixture (50/50 wt%) of zeolite A and Al,Mg-LDH. The Al,Mg-LDH sample was treated at 500 °C in an O_2_ atmosphere and subsequently rehydrated. The Al,Mg-LDH/ZA composites had three treatments: one was performed at 300 °C in a He atmosphere, and two treatments were performed with an O_2_ atmosphere at 300 and 500 °C. In the physical mixture, two treatments were carried out under O_2_ flow at 500 °C and under He flow at 300 °C. Both went through the rehydration process. All samples were also analyzed by energy dispersive spectroscopy (EDS) and scanning electron microscopy (SEM). The results show that the LDH phase in the Al,Mg-LDH/ZA compounds has memory effects, and thus, the compound can be calcined and rehydrated. For the LDH in the composite, the best heat treatment system is a temperature of 300 °C in an inert atmosphere.

## 1. Introduction

Lamellar double hydroxide-like materials (LDHs) can be synthesized in a variety of compositions, the typical one being formed by the hydroxides of Al and Mg forming the lamella and carbonate ions in the interlamellar region, compensating for the structure load [1]. These materials have different physical and chemical properties after the calcination process. They can present high specific areas and can be transformed into homogeneous mixtures of stable oxides presenting small crystal sizes after thermal treatments. However, one of their most interesting properties is called the “memory effect”, which allows the material to reconfigure itself into its original structure after thermal treatment via hydration [2]. Variations in the calcination parameters can also be used to adjust the basic properties of LDH materials. Kwon et al. (2020) [3] studied the memory effect of calcined hydrotalcite in a temperature range of 150–950 °C under an air atmosphere and later rehydrated it with distilled water at 60 °C for 24 h. They sought to evaluate the relationship between heat treatment and rehydration and the basicity of the materials. When comparing the rehydrated material with the calcined starting sample, they observed changes in the basicity. The strength of the basic hydrotalcite sites can be adjusted by the calcination process. High-temperature treatment produces materials with strong basic sites and low-temperature treatment produces weak basic sites. In the case of rehydrated hydrotalcite, both strong and weak basic sites are present. [3]. In a study using the in situ X-ray diffraction technique [4], the formation of a dehydrated phase that could be easily rehydrated was observed, as well as the collapse of the structure above temperatures of 250 °C. It was confirmed that the structure recovery process is slower as the calcination temperature increases.

These materials, with anions compensating for the lamellae, can be used as adsorbents for anionic compounds, including both inorganic [5,6,7,8,9] and organic [10,11,12,13] compounds, and applied to several fields, such as drug delivery [14,15,16,17,18] and effluent and water treatments [19,20,21]. However, they are not efficient for the adsorption of cations. To this end, zeolitic materials have been widely used [22,23,24]. These materials are three-dimensional materials with a pore system formed by the connection of tetrahedral TO_4_ (where T = Si and/or Al) through oxygen atoms. The Al of the network generates a negative charge that is compensated by cations, thus giving the material a cation-exchange property. These materials are used in diverse processes, including adsorption, cation exchange, separation, and catalysis [25,26,27,28,29,30].

Recently, our research group synthesized a composite material based on zeolite A and Al,Mg-LDH for the simultaneous adsorption of cations and anions of produced water [31].

In view of this, this work aimed to study the memory effect of hydrotalcite-type compounds in the composite material to prove its effective regeneration and application in water treatment processes for the simultaneous removal of cations and anions.

## 2. Materials and Methods

### 2.1. Materials

In this study, three samples were analyzed: a sample of Al,Mg-LDH, a physical mixture (50/50 wt%) of zeolite A and Al,Mg-LDH, and the Al,Mg-LDH/ZA composite. This composite is formed by the structures of zeolite A and Al,Mg-LDH. The procedure for synthesizing Al,Mg-LDH was based on the methodology used by (Climent et al., 2004), with Mg/Al = 3. The synthesis of the composite was carried out according to the methodology of [31], wherein the synthetic methodologies of zeolite A and LDH with Al/Mg = 3 ratios were adapted.

### 2.2. In Situ XRD Analysis

The structural property investigation of the samples was performed with in situ XRD experiments performed at the XRD1 beamline at the LNLS-CNPEM. The beamline was operated in the Debye–Scherrer geometry at 12 keV and with a set of 24 K Mythen linear detectors covering 120° in the 2θ range and with a resolution of 0.05° of the FWHM (full width at half maximum). Samples were placed between quartz wool inside quartz capillaries with an internal diameter of 1.5 mm and wall thickness of 0.01 mm. The capillary was connected in a capillary cell that was installed in the 3-circle heavy-duty diffractometer of the beamline. The cell was heated by a hot air blower (FMB Oxford) positioned 3 m from the capillary. The heating rate used in the experiment was 5 °C/min. The temperature inside the sample was previously calibrated with a thermocouple inside the capillary using the same experimental conditions and before the beamtime. The cell inlet was connected to the gas pipeline delivering a controlled flow of He and O_2_ by mass flow meters (10 mL/min). Both could also flow through a saturator with pure H_2_O to carry humidity to the sample (10 mL/min at 25 °C). Diffractograms were collected at 30 s intervals. The rehydration time was defined as 80 min (1.2 h).

The Al,Mg-LDH sample was treated at 500 °C in an O_2_ atmosphere and subsequently rehydrated to collect data regarding the memory effect to be compared to the composite and physical mixture.

Three treatments were carried out on the Al,Mg-LDH/ZA composites. One treatment was performed at 300 °C in a He atmosphere, and two treatments were performed in an oxidizing atmosphere (O_2_) at temperatures of 300 and 500 °C. In the physical mixture, two treatments were carried out under O_2_ flow at a temperature of 500 °C and under He flow at 300 °C. Both went through the rehydration process. At the end of the calcination process, the sample was cooled to room temperature and then rehydrated under H_2_O steam at room temperature. The total rehydration time was 80 min.

At the end of the calcination process, all samples were cooled to room temperature and then rehydrated under H_2_O steam at room temperature. The total rehydration time was 80 min.

### 2.3. Sample Characterization

All samples before and after heat treatment/rehydration were analyzed by energy dispersive spectroscopy (EDS) and scanning electron microscopy (SEM). The analyses were performed using a scanning electron microscope of the ZEISS brand, Auriga model with a FEG type emitter (field emission gun), a voltage of 20 kV, a chemical analysis detector for energy dispersive spectroscopy (EDS) coupled to a mark Bruker and a model xflash detector 410-M.

## 3. Results

The study of the memory effect was performed using a standard Al,Mg-LDH, Al,Mg-LDH/ZA composite, and a physical mixture of Al,Mg-LDH and zeolite A (50 to 50%) to understand the behavior of the HDL/zeolite A composite.

### 3.1. Mg,Al–LDH Memory Effect Study

**Figure 1:**
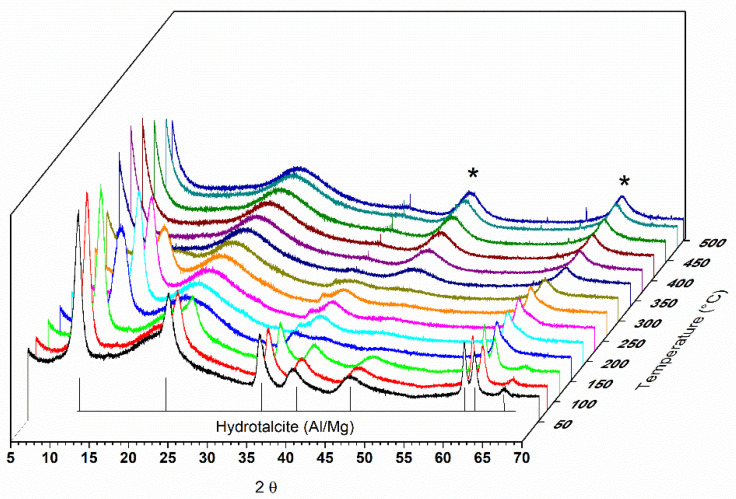
Diffractograms of a sample of lamellar double hydroxide-like materials (LDH) calcined under O_2_ flow at 500 °C. (* magnesium oxide).

The calcination of hydrotalcite-type compounds produces changes in the structure and, consequently, in the sample diffractograms. Figure 1 shows the diffractogram of a sample of hydrotalcite Mg-Al calcined under an oxidizing atmosphere of O_2_ until it reaches a temperature of 500 °C. As the entire study was analyzed by X-ray diffraction in situ, it is possible to observe the structural changes that are occurring in real time.

In the diffractograms shown in Figure 1, it is possible to observe the Al,Mg-LDH phases at the beginning of the treatment, and with the increase in temperature, the transition to another phase is indicated by the presence of two reflections at 2θ = 42.7 and 62.1°. This phase was identified as magnesium oxide (JCPDS 87–0653). The reflections at 2θ = 11.5, 22.9, and 34.5° observed for the hydrotalcite sample at the beginning of the heat treatment refer to the planes (003), (006), and (009) characteristic for this material (JCPDS 41–1428).

During the heat treatment, it is observed that the reflection referring to the plane (003) moves to greater angles, indicating a decrease in the interlamellar space. The basal d spacing value decreases, from 0.77 nm, observed at the beginning of the thermal treatment (50 °C), to a value of 0.66 nm when the treatment temperature is at approximately 300 °C.

**Figure 2:**
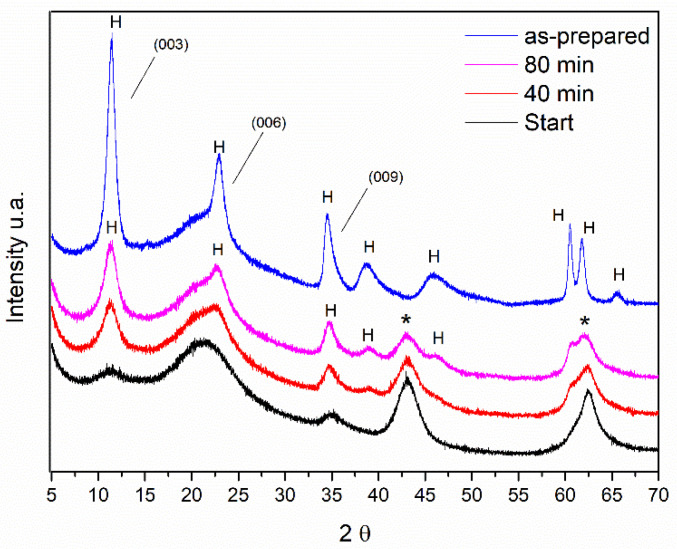
X-ray diffractograms obtained in situ during the rehydration process with steam H_2_O for 80 min of the LDH sample after calcination at 500 °C. The X-ray diffractogram plotted in blue refers to the as-prepared LDH sample, i.e., before calcination. The symbol (*) indicates the reflections belonging to the MgO phase and (H) indicates the reflections referring to the hydrotalcite phase. The plans (003), (006), and (009) characteristic of the LDH phase are also indicated.

Some diffractograms obtained in situ from the rehydration process are shown in Figure 2. For comparison purposes, the sample diffractogram before the calcination process is shown as well.

Most of the diffraction peaks remained during the rehydration process. However, structural changes can be observed following the reflections at 2θ = 11.5°. After 80 min of rehydration, those reflections are more intense and can be observed as other reflections characteristic of LDH but with lower intensity at 2θ = 22.9, 34.5, 38.9, and 46.2°. Comparing the intensities of the reflection at the position 2θ = 11.5° of the samples of Al, Mg-LDH, as-synthesized and after the hydration process, it is observed that this reflection has less intensity. This indicates that the material has not fully returned to its original structure.

**Figure 3:**
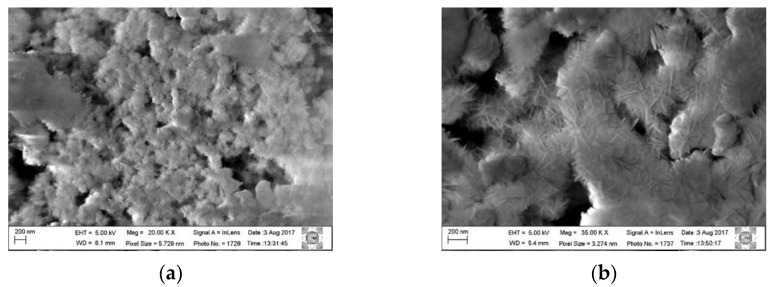

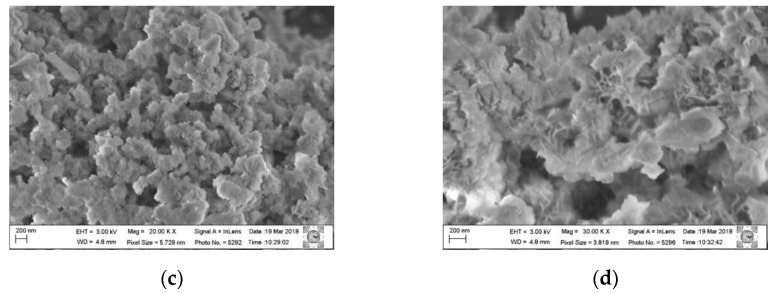
Micrographs of Al,Mg-LDH samples: (**a**,**b**) as-synthesized Al,Mg-LDH sample and (**c**,**d**) calcined sample under O_2_ flow and rehydrated with H_2_O vapor.

This is probably due to the size and organization of the particles. Figure 3 shows the micrographs in different magnifications of the Al,Mg-LDH sample as-synthesized and of the sample after the calcination and hydration process.

**Figure 4:**
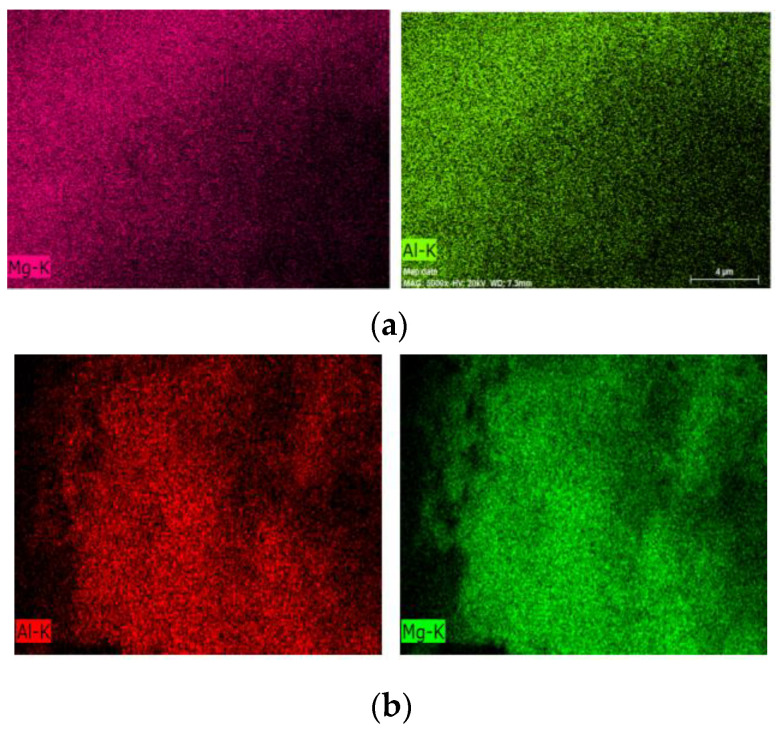
EDS (energy dispersive spectroscopy) LDH images of (**a**) as-synthesized Al,Mg-LDH and (**b**) calcined samples under O_2_ flow and rehydrated with H_2_O vapor.

The morphology presented by the samples remains similar, and it is observed that the synthesized samples have crystal clusters on a nanometric scale; in the starting sample, these crystals are more evenly distributed. In the rehydrated sample, these crystals are less uniform and are more dispersed. In the chemical analysis using EDS (Figure 4), it is observed that the chemical composition remains and that the distribution of Al and Mg is uniform among the particles.

The composition results obtained are shown in Table 1 together with the Mg/Al molar ratio obtained for LDH-type materials before and after heat treatment/rehydration.

### 3.2. LDH/zeolite A Composite Effect Memory Study

**Figure 5:**
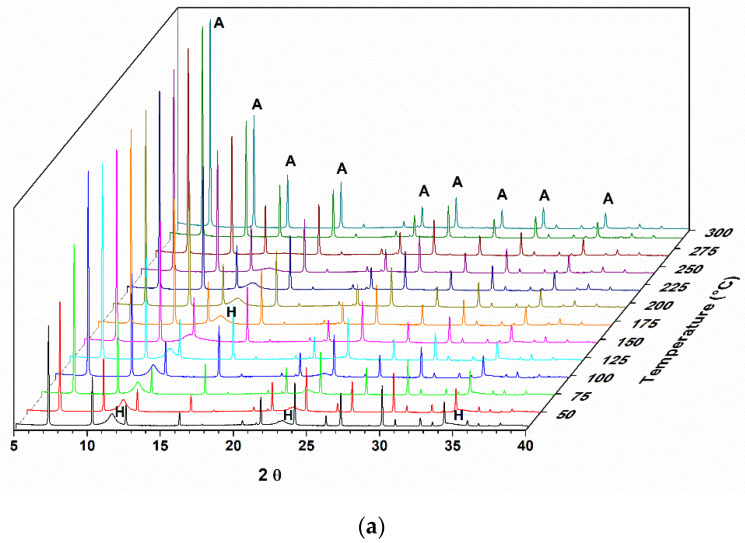

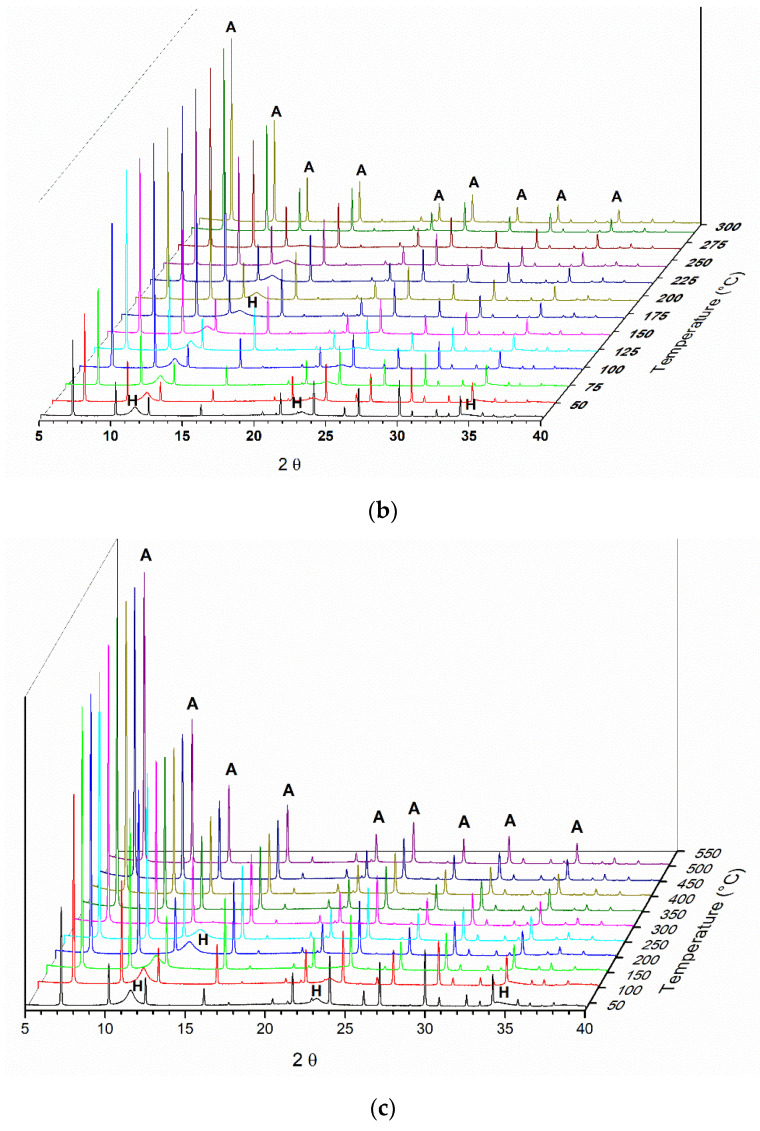
In situ diffractograms of the calcined samples: (**a**) under He flow at 300 °C, (**b**) under O_2_ flow at 300 °C and (**c**) under O_2_ flow at 500 °C. The symbol (A) indicates the reflections belonging to the zeolite A phase and (H) indicates the reflections referring to the hydrotalcite phase. The planes (003), (006), and (009) characteristic of the LDH phase are also indicated.

To investigate the memory effect of the LDH/zeolite A composite, three thermal treatments were carried out. First, the sample was heated to 350 °C under an O_2_ atmosphere (Figure 5a). Second, it was heated using an inert atmosphere with He up to 350 °C (Figure 5b). Then, the sample was exposed to ambient conditions (oxidizing atmosphere) with a flow of O_2_ and ambient temperature up to 500 °C (Figure 5c).

In all three experiments, the X-ray diffraction reflections regarding zeolite A are maintained, with a slight loss in crystallinity, as observed by an increase in the diffraction peak full width at half maximum (FWHM) of the characteristic peaks. In the case of reflections related to LDH, their disappearance is clearly observed as the calcination temperature increases. Due to that, the reflections of zeolite A are much more intense because this phase has larger crystals. It is difficult to observe the two reflections at approximately 40° and 65° referring to the formation of an oxide phase MgO.

The reflection at 2θ = 11.5°, referring to the plane (003) of the LDH phase in the composite, moves to higher values with heating. The behavior is equal to that of the pure LDH sample. This behavior occurs in the diffractograms of the treated samples regardless of the treatment atmosphere and temperature. Separately analyzing a sample diffractogram obtained in each treatment in the temperature range between 235 and 250 °C, it was observed that the basal spacing values for all selected samples was around 0.64–065 nm. These values are consistent with those of pure LDH samples.

**Figure 6:**
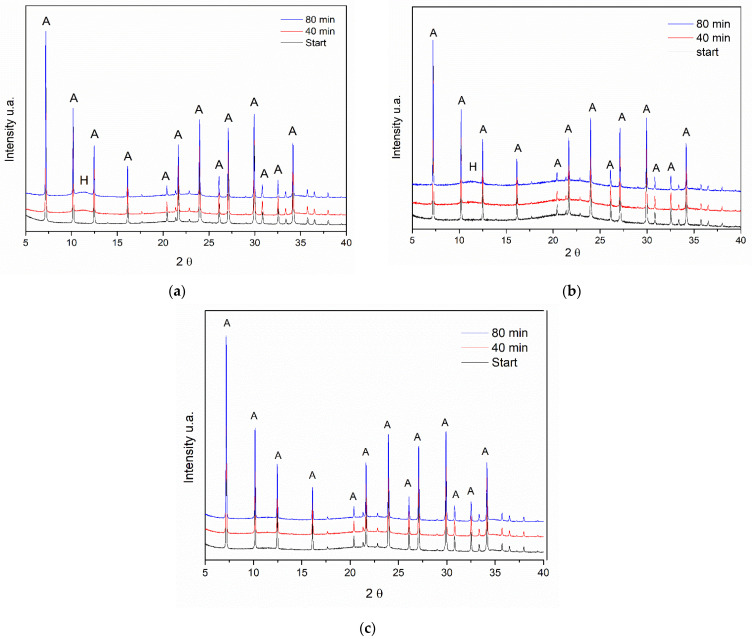
Diffractograms of the samples after rehydration in an atmosphere of H_2_O vapor at room temperature: (**a**) He flow at 300 °C, (**b**) O_2_ flow at 300 °C, and (**c**) O_2_ flow at 500 °C. The symbol (A) indicates the reflections belonging to the zeolite A phase and (H) indicates the reflections referring to the hydrotalcite phase. The plane (003) characteristic of the LDH phase is also indicated in in rehydrated sample.

Following the calcination and cooling process, these samples were rehydrated, and in situ diffractograms were obtained during this process. Figure 6 shows the diffractograms of the rehydration step for the three experiments.

For the composite sample calcined under He flow at 300 °C (Figure 6a) in the range 2θ = 10 − 15°, it is possible to observe the return of the reflection referring to the HDL phase at 2θ = 11.5°. However, as in the sample of pure LDH, the intensity of the reflection related to the plane (003) is exceptionally low. In the sample calcined under the same conditions, it is also possible to see the same O_2_ flow behavior (Figure 6b); however, for the samples calcined under O_2_ flow at 500 °C, the reflection mentioned above is not observed (Figure 6c).

These results indicate that calcination at elevated temperatures (500 °C) hinders the regeneration of the composite.

**Figure 7:**
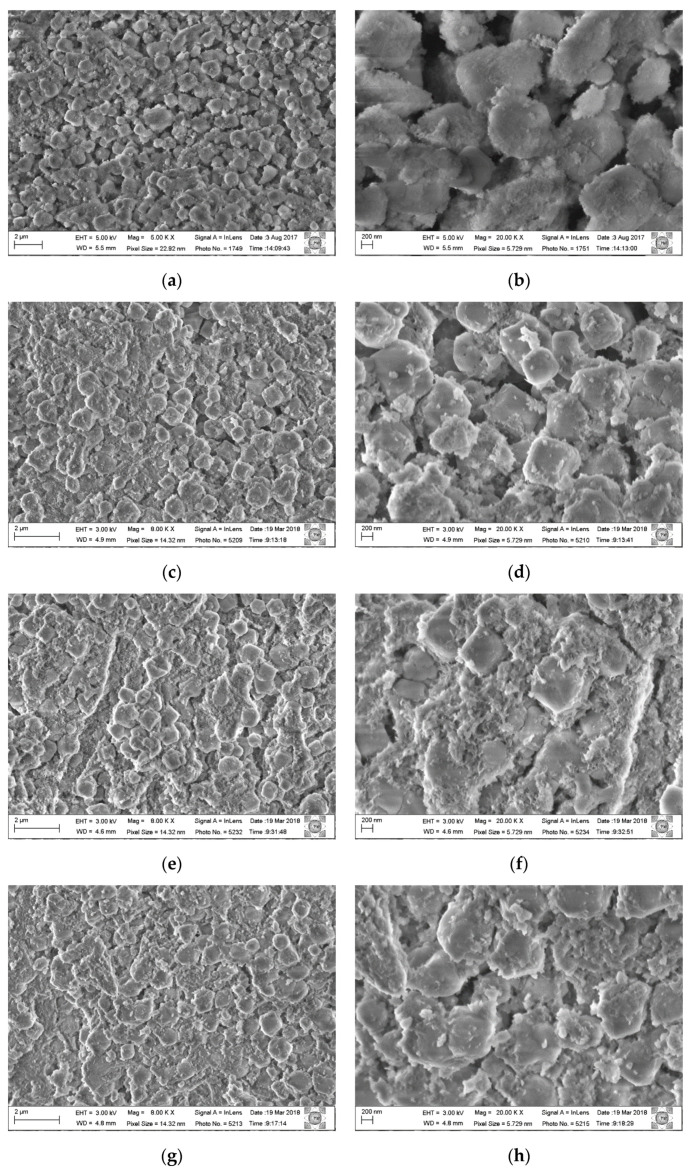
Micrographs of the composite Al,Mg-LDH/ZA, before and after calcination and rehydration processes: (**a**,**b**)as-synthesized composite Al,Mg-LDH/ZA; (**c**,**d**) composite calcined at 300 °C under the flow of He and rehydrated; (**e**,**f**) composite calcined at 300 °C under the flow of O_2_ and rehydrated; (**g**,**h**) composite calcined at 500 °C under the flow of O_2_ and rehydrated.

All composite samples recovered after the hydration process were analyzed by scanning electron microscopy. Figure 7 shows micrographs of the as-synthesized composite Al, Mg-LDH/ZA and composite Al, Mg-LDH/ZA, calcined and rehydrated with different magnifications. In Figure 7a,b, the sample analyzed was the composite as-synthesized, without receiving thermal treatment. Figure 7c,d shows the micrographs of the composite calcined and rehydrated at 300 °C under the flow of He. Figure 7e,f shows the composite calcined and rehydrated at 300 °C under the flow of O_2_. Figure 7g,h shows the composite calcined at 500 °C under the flow of O_2_.

In the composite micrograph, it can be seen that the LDH nanoparticles surround the zeolite crystals. After the calcination process, phase segregation occurs to a greater or lesser degree. Comparing the micrographs of the calcined samples using He and O_2_ at 300 °C, a small segregation is observed between the Al,Mg-LDH/ZA composite phases, but many crystals of zeolite A are clustered with larger particles. It is also observed that the crystals of zeolite A are more rounded and misshapen.

**Figure 8:**
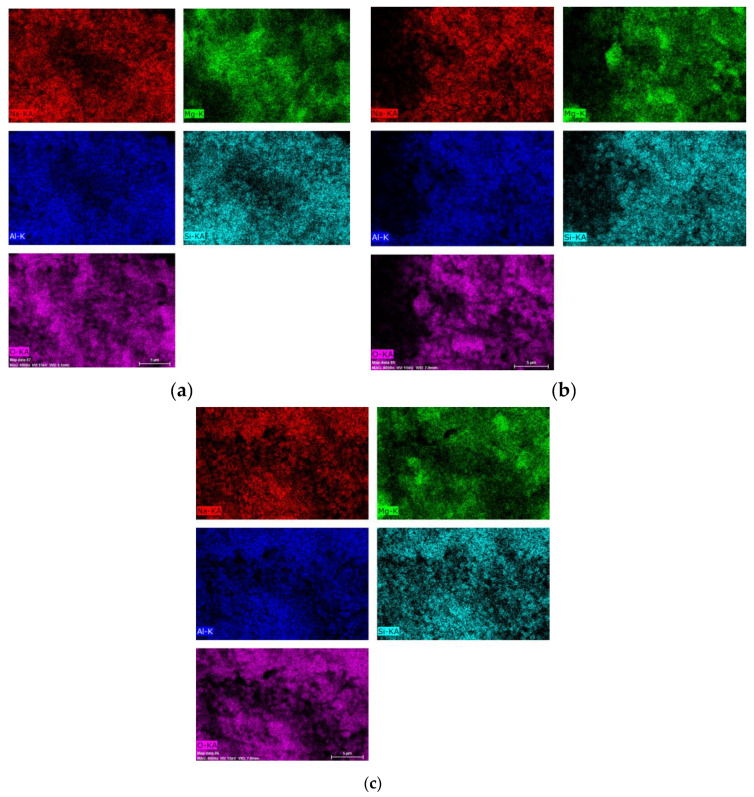
Micrograph of the calcined and rehydrated samples: (**a**) calcination under He flow at 300 °C, (**b**) calcination under O_2_ flow at 300 °C, and (**c**) calcination under O_2_ flow at 500 °C.

Figure 8 presents the results of the chemical analysis obtained for the mapping of the Al,Mg-LDH/ZA composite samples obtained after calcination and subsequent rehydration.

The images show some homogeneity, which shows that the composite maintains its integrity; that is, there are still zeolite A crystals covered by Al,Mg-LDH/ZA. Table 2 shows the values obtained for the chemical composition of the studied samples, as well as the Mg/Al molar ratio obtained for each material.

### 3.3. Physical Mixture of LDH and Zeolite A Memory Effect Study

**Figure 9:**
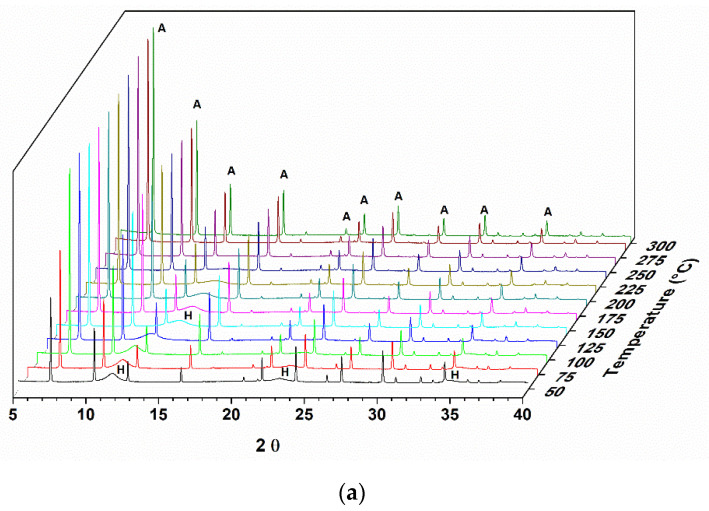

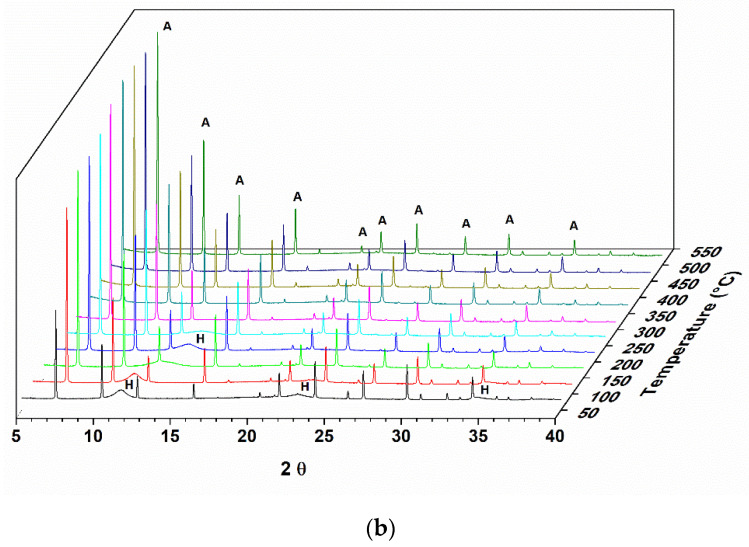
Diffractograms of the physical mixture of 50 wt% LDH and zeolite A: (**a**) calcined under the flow of He and a temperature of 300 °C and (**b**) under the flow of O_2_ and a temperature of 500 °C. The symbol (A) indicates the reflections belonging to the zeolite A phase and (H) indicates the reflections referring to the hydrotalcite phase. The planes (003), (006), and (009), characteristic of the LDH phase, are also indicated.

A 50 wt% physical mixture of LDH and zeolite A was also heat treated under He flow at 300 °C and O_2_ flow at 500 °C. Figure 9 shows the diffractograms for these samples.

**Figure 10:**
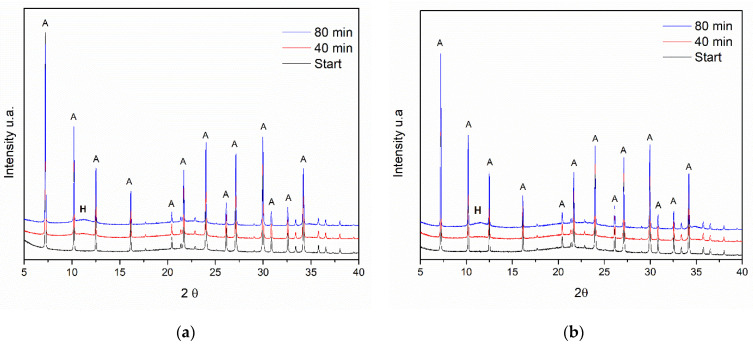
Diffractograms of the Al,Mg-LDH and zeolite A physical mixture rehydrated after (**a**) calcination under He flow and a temperature of 300 °C and (**b**) O_2_ flow and a temperature of 500 °C. The symbol (A) indicates the reflections belonging to the zeolite A phase and (H) indicates the reflections referring to the hydrotalcite phase. The plane (003) characteristic of the LDH phase is also indicated in the rehydrated sample.

The physical mixture presents a behavior similar to the composite (Figure 5) with regard to the diffractograms obtained; at a temperature above 300 °C, it is not possible to observe the presence of the characteristic reflections of LDH. These samples were also rehydrated, and the diffractograms are shown in Figure 10.

The final diffractogram of the rehydrated physical mixture after calcination under He flow at 300 °C is similar to that obtained for the Al,Mg-LDH/ZA composite (Figure 6). The reflection at 2θ = 11.5° is visible but with little intensity. In the case of calcination at 500 °C under O_2_ flow, the signal intensity at 2θ = 11.5° is similar in intensity.

**Figure 11:**
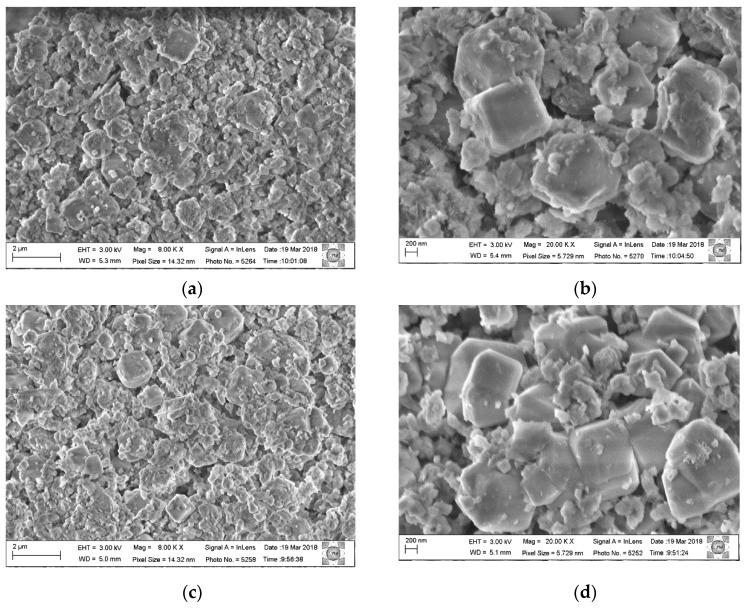
Micrographs of the calcined and rehydrated physical mixture: (**a**,**b**) under He flow and heated to 300 °C, with different magnifications. Micrographs (**c**,**d**) under the flow of O_2_ at 500 °C, with different magnifications.

Micrographs of the physical mixtures calcined and rehydrated under He and O_2_ flows were also obtained and are shown in Figure 11. For each of the calcined and rehydrated samples, two micrographs with different magnifications are presented.

Comparing the images of the physical mixture with the composite samples (Figure 7), it is observed that the phase segregation is much greater with dispersed clusters of LDH and zeolite A crystals. The morphology of the zeolite crystals is closer to that normally found for this structure, demonstrating cubic crystals with beveled edges.

**Figure 12:**
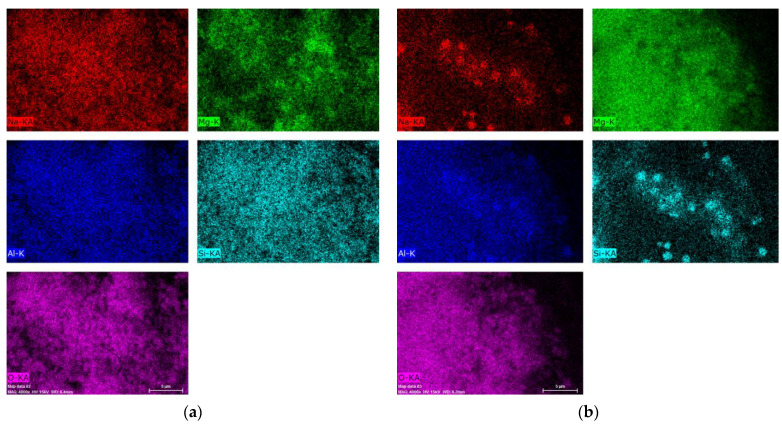
Physical mixture samples obtained after (**a**) calcination under He flow at 300 °C and (**b**) calcination under O_2_ flow at 300 °C. Both samples underwent the rehydration process.

Figure 12 presents the results of the chemical analysis obtained for the mapping of the physical mixture obtained after calcination under He and O_2_ flow and subsequent rehydration.

In Figure 12a, we can see that, among the elements analyzed, Mg has a less uniform distribution with the appearance of some clusters. When we analyze the image in Figure 12b, we can see that Si and Na have a less uniform distribution. In the case of Si, we can observe regions where there are separate zeolite crystals. Table 3 shows the values obtained for the chemical composition of the studied samples as well as the Mg/Al molar ratio obtained for each material.

## 4. Discussion

In evaluating the memory effect of a sample of pure Al,Mg-LDH by in situ X-ray diffraction, it was observed that, after heating to 500 °C with O_2_ flow, it was possible to recover part of the structure. The Al,Mg-LDH samples, as observed in the diffractograms and SEM analysis, show very small crystals and an amount of amorphous material that justifies the profile observed in the diffractogram of the starting sample. As the rehydration process took place in situ and not in solution, this may also have contributed to a reduced organization of the final structure. In the diffractograms of Figure 1, we can observe a displacement of the first reflection until the moment when it disappears.

The displacement of the first peak to values less than 20 was also observed by Hutson and collaborators (2004) [32] in the diffractograms obtained in situ from heat-treated samples over N_2_ flow. This displacement of the signals is related to the removal of H_2_O from the interlayer space. It is observed that, starting from temperatures close to 300 °C, two extended peaks appear, corresponding to the MgO phase. According to the scheme proposed by [3], at a temperature of 200 °C, LDH releases H_2_O molecules, and as the heat treatment progresses, H_2_O molecules are released due to the dehydroxylation of the Al(OH)_3_ groups (270–350 °C). In the range of 350–470 °C, H_2_O is released by the decomposition of the hydroxyl groups of Mg(OH)_2_. From 200 to 600 °C, CO_3_^2−^ bound to Al(OH)_3_ and Mg(OH)_2_ is also released. In addition, Al_2_O_3_ and MgO are formed. From the micrographs shown in Figure 2, it can be seen that, after rehydration, Al,Mg-LDH presents greater agglomeration and less uniformity in the crystals. In the EDS analysis, we observed that the distribution of the elements Al and Mg was slightly less uniform than that observed for as-synthesized Al,Mg-LDH.

The evaluation of the “memory effect” of the composite was evaluated by X-ray diffraction in situ in two different atmospheres, one oxidizing and the other inert. In diffractograms a and b of Figure 5, the displacement of the reflection is observed at approximately 20 = 11.5° for greater angles, indicating the exit of water molecules present between layers. This behavior was not influenced by the type of atmosphere used. However, by submitting the composite to an oxidizing atmosphere (O_2_) at higher temperatures, we observed the same behavior for H_2_O output (Figure 5c). In this way, the Al,Mg-LDH present in the composite behaves similarly when heated as pure LDH does.

After the rehydration process of the composite, the diffractograms (Figure 6) do not show LDH phase reflections with the same intensity as the initial material. We can relate this behavior to the standard sample where the memory effect was not accentuated. Additionally, the fact that the zeolite phase shows very intense signals (very crystalline material) means that the LDH signals cannot be observed very clearly. Nevertheless, it can be observed that the treatment in an inert atmosphere and at a lower temperature favored the restructuring of the material. It should be noted that, in the present experiment, the sample for rehydration was submitted to a humid atmosphere and not directly in H_2_O, which, for complete rehydration, is not the most efficient method.

Using scanning electron microscopy (Figure 7), it is observed that the composite has a morphology that approximates the cubic shape that is characteristic of zeolite A; that is, the LDH phase coats the crystals of zeolite A. With X-ray diffraction, we can follow the evolution of the LDH structure, but only with the use of microscopy can we verify that, with the heating and subsequent rehydration process, Al,Mg-LDH remains adhered to the zeolite crystals. Comparing the micrographs of the composite without heat treatment (Figure 7a) with the treated samples, regions of segregation of the two phases are observed. We can attribute this to the formation of larger LDH particles or even the presence of MgO. This can best be seen when we analyze the distribution of Mg in the post-rehydration materials (Figure 8). Especially for samples heated in an oxidizing atmosphere, rehydrated regions of higher Mg concentration can be observed.

To better understand the process and prove the maintenance of the composite, experiments were carried out with a physical mixture containing 50% LDH and 50% zeolite A. This mixture also underwent heat treatments in He flow at 300 °C and O_2_ flow at 500 °C. The behavior of the physical mixture in the two treatments was similar to that observed for the composite, which was already expected for the XRD analysis. Comparing the diffractograms of the rehydrated composite (Figure 5) with the physical mixture after the rehydration process (Figure 10), it is observed that the behavior was similar. However, in the analysis using scanning electron microscopy (Figure 11) and the distribution of the Mg and Si (Figure 12) in the rehydrated physical mixture, the phases were much more segregated. We can clearly distinguish zeolite A crystals or even their agglomerates.

Comparing the chemical analyses of the composite and the physical mixture after the treatments, it can be observed that there was no significant variation in the Mg/Al ratio, with all presenting values close to three. What is interesting is how the composite had a more homogeneous composition in all experiments, and the atomic percentages of Mg, Si, and Al remained close. In the physical mixture, despite the same conditions (sample holder, preparation, and heating), there is a disparity in the percentages of these elements. That is, in the procedure performed with the physical mixture at 500 °C, a greater amount of LDH was present since the Si percentages are much lower.

For time-optimization of the experiments performed in the synchrotron laboratory, first, the experiments with the composite material were performed using He at 300 °C and then O_2_ at 300 °C (to see the oxidizing atmosphere), and then O_2_ at 500 °C to utilize a hard condition. For the other samples, for LDH only, the hard conditions were utilized to see the memory effect, and for the 50/50 sample (physical mixture), both light conditions (He/300 °C) and hard conditions (O_2_/500 °C) were utilized. Further experiments will be done for all conditions.

## 5. Conclusions

XRD in situ is a very powerful tool to see structural changes and understand the material composite’s behavior under different conditions, using heat and different atmospheres. Specifically, in this work, hard and light conditions of heating under oxidizing and inert atmospheres were studied, followed by a rehydration process.

The LDH phase in the Al,Mg-LDH/ZA compounds has memory effects, and thus, the compound can be calcined and rehydrated. For the LDH in the composite, the best heat treatment system is a temperature of 300 °C in an inert atmosphere (light conditions).

The composite minimizes the effect of variation in composition when used in different processes or treatments. This is because, in the physical mixture, wherein we have completely segregated phases, we can have a greater presence of one phase or another, thus decreasing reproducibility.

The Al,Mg-LDH/ZA composite shows a memory effect in different treatment conditions, showing it to be a very interesting material that can be regenerated without losing properties.

## Figures and Tables

**Table 1:** Chemical analysis of the as-synthesized LDH sample and of the calcined/rehydrated LDH sample.

Compounds	atm% Mg	atm% Al	atm% O	Mg/Al
LDH standard	18	5	77	3.5
LDH O_2_/H_2_O	23	8	69	2.9

**Table 2:** Chemical analysis of the LDH/zeolite A composite after different treatments and rehydration with H_2_O vapor.

Samples	atm% Mg	atm% Al	atm% O	atm% Na	atm% Si	Mg/Al *
Al,Mg-LDH/ZA-He-300 °C	10	12	62	8	8	2.9
Al,Mg-LDH/ZA-O_2_–300 °C	9	12	61	8	9	3.1
Al,Mg-LDH/ZA-O_2_–500 °C	9	11	63	8	8	3.1

* The values of the Mg/Al ratio were calculated based on the assumption that the Si/Al ratio of the zeolite A present is equal to 1.

**Table 3:** Chemical analysis of the LDH and zeolite A physical mixture samples after different treatments and rehydration with H_2_O vapor.

Samples	atm% Mg	atm% Al	atm% O	atm% Na	atm% Si	Mg/Al *
Mix HDL/ZA-He-355 °C	9	12	60	9	9	2.8
Mix. HDL/ZA-O_2_–555 °C	19	8	68	2	2	3.1

* The values of the Mg/Al ratio were calculated based on the assumption that the Si/Al ratio of the zeolite A present is equal to 1.

## Data Availability

The data supporting reported results can be supplied if someone asked for it. Please contact the authors for request.
